# Gut colonization with vancomicyn-resistant *enterococci* among patients with hematologic malignancies

**DOI:** 10.1186/s13099-023-00538-z

**Published:** 2023-03-09

**Authors:** Preslava Mihaylova Hristova, Vladislav Milkov Nankov, Ivaylo Georgiev Hristov, Stefan Venelinov Trifonov, Alexandra Sashova Alexandrova, Hristina Yotova Hitkova

**Affiliations:** 1 Department of Microbiology and Virology, Faculty of Pharmacy, Medical University Pleven, 1 St. Kliment Ohridski Str., 5800 Pleven, Bulgaria; 2 Department of Anatomy, Histology, Cytology and Biology, Medical University Pleven, Pleven, Bulgaria; 3 Section of Hematology, Department of Nephrology, Hematology and Gastroenterology, Medical University Pleven, Pleven, Bulgaria; 4 Department of Medical Microbiology, Medical University of Sofia, Sofia, Bulgaria

**Keywords:** Vancomycin resistance, Hematologic malignancies, Colonization, Risk factors

## Abstract

**Background:**

Vancomycin-resistant enterococci (VRE) are well known agents that colonize the gastrointestinal tract of immunocompromised patients, especially those with hematologic malignancies. The aim of the current study was to determine the incidence of and risk factors for colonization with VRE among patients with hematologic malignancies.

**Materials:**

For a nine-month period, all patients admitted to the Hematology ward at University Hospital in Pleven, Bulgaria who had hematologic malignancy and duration of hospitalization of more than 48 h were screened for colonization with VRE. The data collected from patients and their medical records during the entire hospital stay included: demographic characteristics, clinical information and information about all antimicrobials used. A longitudinal study was used to assesses the risk factors and statistical analysis was performed using SPSS version 27.0.

**Results:**

A total of 119 patients were enrolled in the study. Colonization with VRE was established in 18 of them. One patient carried two species, resulting in a total of 19 VRE: 12 *Enterococcus gallinarum*, 4 *Enterococcus casseliflavus*, 2 *Enterococcus faecium* and 1 *Enterococcus faecalis*. VanA phenotype, with high-level resistance of vancomycin (MIC ≥ 256 μg/ml) and teicoplanin (MIC = 96 μg/ml), was demonstrated by one *E. faecium*, which carried *vanA*. The other *E. faecium* and *E. faecalis* expressed low-level resistance to vancomycin (MICs: 8 μg/ml and 12 μg/ml), susceptibility to teicoplanin (MICs = 0.5 μg/ml) and *vanB* was detected. All *E. gallinarum* and *E. casseliflavus* showed low-level resistance to vancomycin and susceptibility to teicoplanin. *E. gallinarum* strains were positive for *vanC1* and *E. casseliflavus* for *vanC2*. Only two patients were colonized with *vanA* or *vanB* enterococci and the rest 16 were positive for *vanC*. The univariate analysis revealed that patient’s age (70–79 years; *p* = 0.025) and multiple myeloma (*p* = 0.001) are risk factors for VRE acquisition among the investigated patients. In addition, the multivariate analysis confirmed that patient’s age (70–79 years) is an independent risk factor for VRE colonization.

**Conclusions:**

Our results showed that 15.1% of patients with hematologic malignancies were colonized with VRE. There was a distinct prevalence of vanC enterococci. Among the analyzed risk factors, advanced age and multiple myeloma contributed to VRE acquisition.

## Background

The role of vancomycin-resistant enterococci (VRE) as medically important pathogens has significantly increased in the recent years. These bacteria are causative agents of hospital-acquired infections, usually involving the urinary tract, surgical sites and the bloodstream [1]. Also, it is well known that gastrointestinal tract carriage of VRE occurs in critically ill patients, especially those admitted to intensive care units, hematology-oncology and transplantation wards [2].

The frequency of VRE colonization in patients with hematologic malignancies is particularly high [3–5] and geographical differences in VRE spread were described among such patients [6]. Many risk factors have been studied for VRE carriage and the most prominent are: advanced age, prolonged hospital stay, exposure to various antibiotics (e.g., cephalosporins, vancomycin and drugs with activity against anaerobic bacteria), underlying comorbid conditions, exposure to medical devices and contact with other VRE colonized patients [7–12].

Intestinal colonization with VRE has been identified as a risk factor for subsequent blood stream infections (BSIs) [13]. A lot of studies have shown a high prevalence of VRE bacteremia in patients with hematologic malignancies [14, 15]. It is interesting to note that these patients are significantly more susceptible to VRE bacteremia than bacteremia caused by vancomycin-susceptible enterococci [13]. Other risks factors for VRE bacteremia are neutropenia and mucositis. The mucosal compromise due to chemotherapy allows VRE translocation into the bloodstream in patients with malignancies [16]. In addition, inefficient clearance of circulating pathogens due to neutropenia increases the risk of VRE BSIs among colonized patients [13].

The most common VR *Enterococcus* species associated with colonization/infection are *E. faecium* and *E. faecalis*, which carry the *vanA* or *vanB* genes [7]. Although, some authors have reported high prevalence of intestinal colonization with low-level vancomycin resistant *E. gallinarum* and *E. casseliflavus* [17], which are rare causative agents of nosocomial infections [18–21].

Despite many reports for VRE acquisition worldwide, the data about the VRE carriage in hospitalized patients in our country are limited. Therefore, the purpose of our study was to determine the incidence of and risk factors for colonization with VRE among patients with hematologic malignancies.

## Methods

### Study design

This study was conducted in the Hematology ward, comprising 46 beds at the University Hospital “Dr. George Stranski” in Pleven, Bulgaria, a tertiary care hospital with 950 beds.

The Hematology ward is a specialized unit for treatment of patients with hematological and hemato-oncological diseases, but without transplant recipients. All patients admitted between May 1st, 2018 and January 31st, 2019, who had hematologic malignancy without a prior history of hospitalization/intervention in the last 30 days and duration of hospitalization of more than 48 h, were enrolled in the study. Written consent was obtained from each patient in accordance with the rules of Hospital Ethics Committee.

The data collected from patients and their medical records during the entire hospital stay included: demographic characteristics (sex and age), clinical information (primary hematologic disease, length of stay in the Hematology ward, co-morbidities, obesity, corticosteroid use, chemotherapy, use of medications prior the hospital admission) and information about all antimicrobials used within the previous 30 days before hospital admission and during the current hospital stay. Epidemiological data related to housing, environmental or work-related factors was collected for all colonized patients.

To determine the intestinal colonization with VRE, fecal samples were taken within the first 48 h of hospital stay, on the 5th day after admission and then on a weekly basis until discharge or death. VRE colonization in each patient without a prior history of hospitalization/intervention in the last 30 days and with a positive initial (in the first 48 h) fecal culture was considered to be community-acquired. All patients with a positive VRE stool culture and without VRE infection or other infections were defined as VRE colonized group. The non-VRE colonized group included patients without detected VRE colonization or infections.

### Bacterial cultures

Samples were directly inoculated on Brilliance VRE agar (*Oxoid, United Kingdom*), as well as into bile aesculin azide broth (*Liofilchem, Italy*) supplemented with 6 μg/ml vancomycin (BEAV broth). The inoculated culture media were incubated aerobically at 37 °C and were examined for growth at 24 h and 48 h. The identification of the enterococci growing on Brilliance VRE agar was based on the observation of appropriately colored colonies – indigo to purple for *E. faecium* and light blue for *E. faecalis*. Growing colonies were transferred to a 5% Blood agar plate (BAP) and re-incubated for 24 h.

The positive BEAV broths that developed black color were subcultured to 5% BAP and chromID CPS Elite (*bioMérieux, France*) and incubated for an additional 24 h. All suspected VRE, isolated from Brilliance VRE agar and BEAV broth were identified using Vitek 2 Compact (*bioMérieux, France*). In cases of low-level discrimination between *E. gallinarum* and *E. casseliflavus*, motility and pigment production tests were also done.

### Antimicrobial susceptibility testing and detection of *vanABCDMN* genes of resistance

Minimum inhibitory concentrations (MICs) to ampicillin, gentamicin, vancomycin, teicoplanin, ciprofloxacin, tigecycline, linezolid, daptomycin and quinupristin/dalfopristin were examined by *E*-test (Liofilchem, Italy). MICs results were interpreted according to the recommendations of The European Committee on Antimicrobial Susceptibility Testing (EUCAST Breakpoint tables for interpretation of MICs and zone diameters, version 11.0, 2021, http://www.eucast.org/clinical_breakpoints/). The Clinical and Laboratory Standards Institute (CLSI) guidelines, 2021, https://clsi.org/standards/ were used to interpret the MICs for daptomycin. The presence of *vanABCDMN* genes was investigated by colony multiplex PCR assay using the primer sequences and PCR protocol described by Nomura et al. [22]. Briefly, a modified PCR mix for detection of the investigated genes was applied containing 0.4 µM (each) primer, 200 µM (each) dNTP, 1 U of Taq (Canvax, Spain), 1X reaction buffer, 2.5 mM MgCl_2_, ultrapure PCR H_2_O and 10 ng DNA template to a final volume of 20 µL. The PCR thermal conditions consisted of initial denaturation (94 °C for 4 min), followed by 30 cycles of denaturation (94 °C for 30 s), annealing (62 °C for 35 s) and extension (68 °C for 1 min), with a single final extension of 7 min at 68 °C. The amplified PCR products were analyzed by capillary electrophoresis.

### Statistical analysis

Statistical analysis of the risk factors for VRE colonization was performed using SPSS version 27.0 (Armonk, NY: IBM Corp.). Categorical variables were analyzed using the Chi-square test of independence or Fisher’s exact test when the expected values were below 5. Odds ratios (OR) and 95% confidence intervals (CI) were determined to assess the strength of any associations. Evaluation of the risk factors for VRE colonization was performed using univariate logistic regression analysis. Multiple logistic regression was performed for all significant variables in the univariate analysis to determine what risk factors were independently associated with the development of VRE colonization. Statistical significance was defined as *p* < 0.05.

## Results

A total of 119 patients with hematologic malignancies were enrolled in this investigation. The median age of the studied participants was 67 (range: 20–88) years, 69 (58%) were male and 50 (42%) were female. The mean length of hospital stay was 9 days (range: 6–46 days). The most frequent reason for admission was cytotoxic chemotherapy (n = 53, 44.5%). The most frequent underlying malignancies were myelodysplastic syndrome (n = 28), non-Hodgkin’s lymphoma (n = 26), multiple myeloma (n = 20) and chronic lymphocytic leukemia (n = 17). Overall, 256 fecal samples were collected with a mean of 2.2 samples per patient (range: 1 to 7). The same microorganism was detected in all patients with two or more positive cultures.

Intestinal colonization with VRE was observed in 18 (15.1%) out of 119 screened patients. One patient was a carrier of two strains (1 VR *E. faecium* and 1 VR *E. faecalis)*, resulting in a total of 19 VRE isolates: 16 with VanC phenotype (12 *E. gallinarum* and 4 *E. casseliflavus*), 2 *E. faecium* and 1 *E. faecalis*.

Antimicrobial susceptibility profiles of the isolated VRE and their *van* genes are presented in Table 1. The results showed that only one *E. faecium* isolate demonstrated a VanA phenotype with high-level resistance to vancomycin (MIC ≥ 256 μg/ml) and teicoplanin (MIC = 96 μg/ml), as well as ampicillin (MIC ≥ 256 μg/ml), gentamicin (MIC ≥ 1024 μg/ml), ciprofloxacin (MIC ≥ 32 μg/ml) and quinupristin/dalfopristin (MIC ≥ 32 μg/ml). The VanA phenotype of this strain was verified by PCR detection of the *vanA* gene. The second *E. faecium* and *E. faecalis* expressed low-level resistance to vancomycin with MICs of 8 μg/ml and 12 μg/ml, respectively, and susceptibility to teicoplanin (MICs = 0.5 μg/ml). *E. faecium* was resistant to ampicillin (MIC ≥ 256 μg/ml) and ciprofloxacin (MIC ≥ 32 μg/ml), while *E. faecalis* was only highly resistant to gentamicin (MIC ≥ 1024 μg/ml). The *vanB* gene was detected in the two isolates. All *E. gallinarum* and *E. casseliflavus* isolates showed low-level resistance to vancomycin (MICs: 4–16 μg/ml), susceptibility to teicoplanin (MICs: 0.5–1.0 μg/ml) and to the rest of the tested antimicrobial agents. Furthermore, the *E. gallinarum* and *E. casseliflavus* strains carried *vanC1* and *vanC2* genes, respectively.

**Table 1:** Antimicrobial susceptibility profiles and *van* genes in VRE isolates

Species	№ of isolates	*Van* genes	MICs or MIC ranges (μg/ml)								
			AMP	GEN	VAN	TEC	CIP	LZD	TGC	DAP	Q/D
*E. faecium*	1	*vanA*	≥ 256	≥ 1024	≥ 256	96	≥ 32	3	0.047	2	≥ 32
*E. faecium*	1	*vanB*	≥ 256	8	8	0.50	≥ 32	2	0.064	1	2
*E. faecalis*	1	*vanB*	0.25	≥ 1024	12	0.50	2	3	0.125	0.75	3
*E. gallinarum*	12	*vanC1*	1–2	0.75–4	4–16	0.5–1	1–4	1–3	0.032–0.19	0.38–2	–
*E. casseliflavus*	4	*vanC2*	0.5–1	0.5–2	4–12	0.5–1	1–4	1–3	0.064–0.125	0.38–1	–

*AMP* ampicillin, *GEN* gentamicin, *VAN* vancomycin, *TEC* teicoplanin, *CIP* ciprofloxacin, *LZD* linezolid, *TGC* tigecycline, *DAP* daptomycin, *Q/D* quinupristin/dalfopristin

Only two patients were colonized with *vanA* or *vanB* enterococci. The first one, an 85-year-old woman with multiple myeloma, was a carrier of *vanA E. faecium* and *vanB E. faecalis*. The second patient, a 75-year-old man with chronic lymphocytic leukemia, was colonized with *vanB E. faecium*.

The results from the univariate statistical analysis of the risk factors for VRE colonization are shown in Table 2. The patients included in the analysis were divided in two groups – VRE colonized and non-VRE colonized. Of all 18 VRE-positive patients, 13 were included in the first group, because their VRE colonization was hospital-acquired. The remaining 5 patients had positive cultures for *vanC*-carring enterococci within the first 48 h of the hospital stay and they were excluded. None of the VRE colonized patients had acquired other infection during the hospital stay. Of all 101 non-VRE colonized patients, 5 developed an infection and were excluded from the statistical analysis. Comparison between 13 patients with hospital-acquired colonization and 5 patients with community-acquired colonization is presented in Table 3.

**Table 2:** Risk factors for colonization with VRE in hospitalized patients with hematologic malignancies

Risk factors	Frequency (%) or mean ± SD			Univariate analysis	
	VRE colonized	non-VRE colonized	Total	OR (95% CI)	*p*-value
Demographics					
Age (years)^a^	74.31 ± 6.58	65.52 ± 13.37	–	3.697 (1.114–12.264)	0.025
Gender: male: female	9 (69.23): 4 (30.77)	54 (65.25):42 (43.75)	109 (100)	0.571 (0.165–1.984)	0.374
Mean length of stay (days)	11.15 ± 4.705	8.57 ± 5.11	–	1.524 (-5.602–0.440)	0.093
Primary hematologic disease (%)					
Myelodysplastic syndrome	3 (23.08)	20 (20.83)	23 (21.1)	1.140 (0.287–4.536)	0.852
Non-hodgkin lymphoma	0	25 (26.04)	25 (22.94)	0.1 (0.01–1.81)	0.081
Multiple myeloma	7 (53.85)	13 (13.54)	20 (18.35)	7.449 (2.161–25.669)	0.001
Chronic lymphocytic leukemia	2 (15.38)	13 (13.54)	15 (13.76)	1.161 (0.231–5.843)	0.856
Hodgkin Lymphoma	0	9 (9.38)	9 (8.26)	0.34 (0.02–6.21)	0.249
Chronic erythremia	0	4 (4.17)	4 (3.67)	0.76 (0.04–14.94)	0.453
Acute myeloblastic leukemia	1 (7.69)	4 (4.17)	5 (4.59)	1.917 (0.198–18.596)	0.569
Acute lymphoblastic leukemia	0	3 (3.13)	3 (2.75)	0.99 (0.05–20.23)	0.518
Acute myelomonocytic leukemia	0	2 (2.08)	2 (1.83)	1.4 (0.06–30.75)	0.599
Acute promyelocytic leukemia	0	1 (1.04)	1 (0.92)	2.36 (0.09–60.87)	0.712
Hairy-cell leukemia	0	1 (1.04)	1 (0.92)	2.36 (0.09–60.87)	0.712
Chronic monocytic leukemia	0	1 (1.04)	1 (0.92)	2.36 (0.09–60.87)	0.712
Underling diseases (%)					
Chronic heart failure	9 (69.23)	48 (50)	53 (48.62)	2.250 (0.649–7.805)	0.193
Chronic obstructive pulmonary disease	1 (7.96)	10 (10.42)	11 (10.09)	0.717 (0.084–6.107)	0.760
Chronic renal failure	2 (15.38)	7 (7.29)	9 (8.26)	2.312 (0.426–12.550)	0.332
Insulin-dependent diabetes mellitus	5 (38.46)	18 (18.75)	23 (21.1)	2.708 (0.792–9.259)	0.112
Obesity	2 (15.38)	14 (14.58)	16 (14.68)	1.065 (0.213–5.326)	0.939
Chemotherapy	8 (61.54)	45 (46.88)	53 (48.62)	1.813 (0.553–5.943)	0.326
Corticosteroids use	7 (53.85)	39 (40.63)	46 (42.2)	1.705 (0.532–5.461)	0.369
Medications prior the hospital admission (%)					
Cardiovascular medications	9 (69.23)	46 (47.92)	55 (50.46)	2.446 (0.705–8.485)	0.149
Endocrine medications	6 (46.15)	20 (20.83)	26 (23.85)	2.375 (0.700–8.053)	0.156
COPD medications	1 (7.96)	10 (10.42)	11 (10.09)	0.717 (0.084–6.107)	0.760
Nonsteroids	2 (15.38)	14 (14.58)	16 (14.68)	1.065 (0.213–5.326)	0.939
Other medications^b^	1 (7.96)	3 (3.13)	4 (3.67)	2.583 (0.248–26.863)	0.403
Antibiotic use in last 30 days and during the current hospital stay (%)					
3rd generation cephalosporins	1 (7.96)	3 (3.13)	4 (3.67)	2.583 (0.248–26.863)	0.403
Fluroquinolones	1 (7.96)	2 (2.08)	3 (2.75)	3.917 (0.330–46.514)	0.319
Macrolides	0	4 (4.17)	4 (3.67)	0.76 (0.04–14.94)	0.453
Antifungal drugs	0	2 (2.08)	2 (1.83)	1.4 (0.06–30.75)	0.599

SD standard deviation, *OR* odds ratio, *CI* confidence interval

^a^Age (years) For the goals of statistical analysis, the studied 96 patients were divided in the followed 7 age groups: 18–29; 30–39; 40–49; 50–59; 60–69; 70–79; 80–89. Statistical significance was found only between the presence of colonization and the age of patients 70–79 years (*p* = 0.025)

^b^Other medications (antiallergic drugs: n = 3; antidepressants: n = 1)

**Table 3:** Comparison between the patients with community-acquired and hospital-acquired colonization with VRE

Characteristics	VRE colonized patients	
	Hospital-acquired	Community-acquired
Demographics		
Mean age (years)	74	73
Gender: male: female	9:4	4:1
Primary hematologic disease (n)		
Myelodysplastic syndrome	3	3
Non-hodgkin lymphoma	0	1
Multiple myeloma	7	0
Chronic lymphocytic leukemia	2	1
Underling diseases (n)		
Chronic heart failure	9	3
Chronic obstructive pulmonary disease	1	1
Chronic renal failure	2	0
Insulin-dependent diabetes mellitus	5	1
Obesity	2	0
Chemotherapy	8	0
Corticosteroids use	7	2
Medications prior the hospital admission (н)		
Cardiovascular medications	9	3
Endocrine medications	6	2
COPD medications	1	1
Nonsteroids	2	2
Other medications^a^	1	0
Antibiotic use in last 30 days and during the current hospital stay (n)		
3rd generation cephalosporins	1	2
Fluroquinolones	1	0
Carbapenems	0	1
Vancomycin	0	1
Metronidazole	0	1

*N* number of patients

^a^Other medications (antiallergic drugs: n = 1)

The data revealed that age of the patients in the range from 70 to 79 years (*p* = 0.025) and multiple myeloma (*p* = 0.001) were risk factors which were significantly associated with VRE intestinal acquisition. In the multivariate analysis, patient’s age (70–79 years) was confirmed as an independent risk factor for VRE colonization.

## Discussion

According to the Center for Disease Control and Prevention (CDC) and the Society for Healthcare Epidemiology of America (SHEA), active screening of hospitalized patients for VRE carriage is necessary [23–25]. Thus, it has been suggested as an effective infection prevention and control measure.

In most of studies, the majority of VRE intestinal isolates are *E. faecium* and *E. faecalis*, but we detected a low ratio of these species among patients with hematologic malignancies in our hospital. Only two patients (1.7%) were colonized with VR *E. faecium* and/or VR *E. faecalis*, whereas vanC enterococci were found in 13.4% of the patients. The detailed epidemiological investigation of the colonized patients did not reveal any housing, environmental or work-related factors leading to higher likelihood of colonization with *E. gallinarum* and *E. casseliflavus* versus VR *E. faecium* and/or VR *E. faecalis* species. Our data correlate with the study of Batistao et al. [17], who demonstrated that 77 out of 78 VRE colonized patients were positive for vanC enterococci. Tresoldi et al. [26] also published similar results – 7 out of 9 colonized patients were carriers of *E. gallinarum* or *E. casseliflavus* strains. Maschieto et al. [27] did not isolate either VR *E. faecium* or VR *E. faecalis*, but they showed a high rate of *vanC-*carring enterococci – *E. gallinarum* (23.7%) and *E. casseliflavus*/*E. flavescens* (5.2%). In contrast, Gedik et al. [5] observed that a total of 50 (39.8%) patients carried VR *E. faecium* or VR *E. faecalis*. Other investigators [28] established that 72 (31.4%) children admitted in hematology-oncology wards were carrying VRE and the number of VR *E. faecium* isolates was significantly higher than vanC enterococci. Suzuki et al. [29] investigated the epidemiology of BSIs for a 10-year period in Japan and found that 9% and 2.7% of them were caused by *E. casseliflavus*/*E. flavescens* and *E. gallinarum*, respectively. All these data testify to the increasing number of patients infected/colonized with vanC enterococci.

In the current study, we observed high diversity of VRE genes among the intestinal enterococcal isolates with a higher proportion of *vanC1* gene (63.2%), followed by *vanC2* gene (21%), *vanB* gene (10.5%), and *vanA* gene (5.3%). Although all *vanC*-carrying enterococci (n = 16, 84.2%) displayed a classical VanC phenotype, they can be reservoirs of resistance genes. Corso et al. [30] reported 14 intestinal *E. gallinarum* isolates, which carried dual *vanC1* and *vanA* genes. Shirano et al. described 98 *E. gallinarum* intestinal isolates, from which 88 were positive for *vanA* and 10—for *vanB*. Also, there are evidence for carriage of *vanC1*, *vanA* and *vanB* cassette by *E. gallinarum* [31].

Many risk factors, which can contribute to acquisition of VRE in hematologic malignancy patients, were evaluated in the current study. Analysis of our results showed an association between advanced patient’s age (70–79 years; *p* = 0.025) and fecal carriage of VRE. A correlation between the age of patients and VRE colonization was also found by Metallidis et al. [10], whereas some other studies found no association between the two variables [4, 11, 17, 32]. We also found that patients who suffer from multiple myeloma were most commonly colonized with VRE (*p* = 0.001). The multivariate analysis identified only the advanced age of the patient as an independent risk factor for VRE acquisition. This result was expected, because the prevalence of multiple myeloma increases with age (in patients over the age of 60 years), while most of the other discussed malignancies are more prevalent in younger/middle age. The studies by Mioljevic et al. [12] and Worth et al. [33] demonstrated significantly high prevalence of VRE carriage in patients with acute myeloid leukemia (AML), who usually have a longer length of hospital stay. In our study the evaluated number of patients with AML was small and no one acquired fecal VRE.

A variety of variables were identified as significant factors for VRE acquisition among different groups of immunocompromised patients [11, 17, 34]. Suntharam et al. [11] found that prolonged length of stay, prior hospitalization, previous admission in intensive-care units and amikacin administration played a role in VRE colonization among hematology-oncology patients. In other study, nephropathy, prior antibiotic use and carbapenem administration were identified as risk factors for VRE acquisition among critically ill patients [17]. Papadimitriou-Olivgeris et al. [34] reported the quinolone use, the number of positive VRE patients in nearby beds, the chronic renal failure and the chronic obstructive pulmonary disease as risk factors for VRE colonization during hospital stay in intensive care units. In our study, there was not significant association between gender; mean length of hospital stay; underlying disease; antibiotic use; other types of medications and VRE acquisition.

Intestinal colonization with VRE precedes and plays a key role in the pathogenesis of BSIs in patients with malignancies. The data in the literature showed that the individuals who carried VRE had 24 times higher risk for developing bacteremia with these microorganisms in comparison to the non-colonized patients [6]. Study by Kara et al. [3] founded that among 193 colonized patients, 3 developed bacteremia caused by VRE and 1 of these patients have been under intensive chemotherapy. In our study, 8 of the colonized patients (6 with vanC enterococci and 2 with *E. faecium* and/or *E. faecalis*) were admitted to the hospital due to the need of a chemotherapy course. Although that the high dose chemotherapy promotes VRE-related BSI, none of the patients developed an infection. During the study period in two other hospital wards (Surgery and Intensive Care Unit) there were 3 cases of *vanC* enterococcal infections (2 wound infections and 1 urinary tract infection) without bacteremia.

It is interesting to note that only 13 (11.93%) out of 109 patients included in the study had used antibiotics in the last 30 days and during the current hospital stay. In contrast to our findings was a study by Mioljevic et al. [12] in which only 11 (15.4%) patients did not receive any antibiotic during the hospitalization. In the present study, the patient caring *vanA E. faecium* and *vanB E. faecalis* was treated with third-generation cephalosporin during the hospital stay, whereas the patient with *E. gallinarum* was on therapy with ciprofloxacin prior to hospitalization. In many studies, treatment with these antibiotics was determined as a risk factor for colonization with VRE [7, 34]. Due to this reason, we could suggest that consumption of third-generation cephalosporins and fluoroquinolones might have contributed for the acquisition of intestinal VRE in these patients.

## Conclusions

To our knowledge, this is the first report from Bulgaria which investigated the prevalence of VRE colonization among patients with hematologic malignancies. Intestinal VRE carriage was identified in 15.1% of the patients with a significant prevalence of vanC enterococcal isolates. Among the analyzed risk factors, advanced age and multiple myeloma contributed to VRE acquisition.

## Data Availability

All data generated and analyzed during this study are included in this published article.

## Abbreviations

**BSIs**: Blood stream infections
**CDC**: Center for disease Control and prevention
**CI**: 95% confidence interval
**CLSI**: Clinical and Laboratory Standards Institute
**EUCAST**: The European Committee on Antimicrobial Susceptibility testing
**MIC**: Minimum inhibitory concentration
**OR**: Odds ratio
**PCR**: Polymerase chain reaction
**SHEA**: Society for Healthcare Epidemiology of America
**VRE**: Vancomycin-resistant enterococci

## References

[1] Weiner-Lastinger LM, Abner S, Edwards JR, Kallen AJ, Karlsson M, Magill SS (2020). Antimicrobial-resistant pathogens associated with adult healthcare-associated infections: summary of data reported to the national healthcare safety Network, 2015–2017. Infect Control Hosp Epidemiol.

[2] Yoon YK, Lee SE, Lee J, Kim HJ, Kim JY, Park DW (2011). Risk factors for prolonged carriage of vancomycin-resistant *Enterococcus* faecium among patients in intensive care units: a case-control study. J Antimicrob Chemother.

[3] Kara A, Devrim I, Bayram N, Katipoglu N, Kıran E, Oruc Y (2015). Risk of vancomycin-resistant enterococci bloodstream infection among patients colonized with vancomycin-resistant enterococci. Braz J Infect Dis.

[4] Ford CD, Lopansri BK, Haydoura S, Snow G, Dascomb KK, Asch J (2015). Frequency, risk factors, and outcomes of vancomycin-resistant *Enterococcus* colonization and infection in patients with newly diagnosed acute leukemia: different patterns in patients with acute myelogenous and acute lymphoblastic leukemia. Infect Control Hosp Epidemiol.

[5] Gedik H, Yildırmak T, Simsek F, Kanturk A, Arica D, Aydin D (2014). Vancomycin-resistant enterococci colonization and bacteremia in patients with hematological malignancies. J Infec in Dev Ctries.

[6] Alevizakos M, Gaitanidis A, Nasioudis D, Tori K, Flokas ME, Mylonakis E (2016). Colonization with vancomycin-resistant enterococci and risk for bloodstream infection among patients with malignancy: a systematic review and meta-analysis. Open Forum Infect Dis.

[7] Mazuski JE (2008). Vancomycin-resistant *Enterococcus*: risk factors, surveillance, infections, and treatment. Surg Infect.

[8] Ziakas PD, Thapa R, Rice LB, Mylonakis E (2013). Trends and significance of VRE colonization in the ICU: a meta-analysis of published studies. PLoS ONE.

[9] Pan SC, Wang JT, Chen YC, Chang YY, Chen ML, Chang SC (2012). Incidence of and risk factors for infection or colonization of vancomycin-resistant enterococci in patients in the intensive care unit. PLoS ONE.

[10] Metallidis S, Chatzidimitriou M, Tsona A, Bisiklis A, Lazaraki G, Koumentaki E (2006). Vancomycin-resistant enterococci, colonizing the intestinal tract of patients in a university hospital in greece. Braz J Infect Dis.

[11] Suntharam N, Lankford MG, Trick WE, Peterson LR, Noskin GA (2002). Risk factors for acquisition of vancomycin-resistant *enterococci* among hematology-oncology patients. Diagn Microbiol Infect Dis.

[12] Mioljevic V, Markovic-Denic L, Vidovic A, Jovanovic M, Tosic T, Tomin D (2013). Risk factors for vancomycin-resistant *Enterococcus* colonization in hematologic patients. Vojnosanit Pregl.

[13] Peel T, Cheng AC, Spelman T, Huysmans M, Spelman D (2012). Differing risk factors for vancomycin-resistant and vancomycin-sensitive enterococcal bacteraemia. Clin Microbiol Infect.

[14] Bossaer JB, Hall PD, Garrett-Mayer E (2011). Incidence of vancomycin-resistant enterococci (VRE) infection in high-risk febrile neutropenic patients colonized with VRE. Support Care Cancer.

[15] Weinstock DM, Conlon M, Iovino C, Aubrey T, Gudiol C, Riedel E (2007). Colonization, bloodstream infection, and mortality caused by vancomycin-resistant *Enterococcus* early after allogeneic hematopoietic stem cell transplant. Biol Blood Marrow Transpl.

[16] Kuehnert MJ, Jernigan JA, Pullen AL, Rimland D, Jarvis WR (1999). Association between mucositis severity and vancomycin-resistant enterococcal bloodstream infection in hospitalized cancer patients. Infect Control Hosp Epidemiol.

[17] da Batistao DW, Gontijo-Filho F, Conceicao PP, Oliveira N, de Ribas AG (2012). Risk factors for vancomycin-resistant enterococci colonisation in critically ill patients. Mem Inst Oswaldo Cruz.

[18] Reid KC, Cockerill FR, Patel R (2001). Clinical and epidemiological features of *Enterococcus*
*casseliflavus/flavescens* and *Enterococcus*
*gallinarum* bacteremia: a report of 20 cases. Clin Infect Dis.

[19] Narciso-Schiavon JL, Borgonovo A, Marques PC, Tonon D, Bansho ETO, Maggi DC (2015). *Enterococcus*
*casseliflavus* and *Enterococcus*
*gallinarum* as causative agents of spontaneous bacterial peritonitis. Ann Hepatol.

[20] Vasilakopoulou A, Vourli S, Siafakas N, Kavatha D, Tziolos N, Pournaras S (2020). *Enterococcus*
*casseliflavus* bacteraemia in a patient with chronic renal disease. Infect Dis Rep.

[21] Amaro P, Ferreira J, Viegas R, Cardoso A, Correia J, Maurício H (2021). Multifocal joint infection caused by *Enterococcus*
*gallinarum*. Mod Rheumat Case Reports.

[22] Nomura T, Hashimoto Y, Kurushima J, Hirakawa H, Tanimoto K, Zheng B (2018). New colony multiplex PCR assays for the detection and discrimination of vancomycin-resistant enterococcal species. J Microbiol Methods.

[23] Siegel JD, Rhinehart E, Jackson M, Chiarello L (2007). Management of multidrug-resistant organisms in health care settings, 2006. Am J Infect Control.

[24] Shay DK, Goldmann DA, Jarvis WR (1995). Reducing the spread of antimicrobial-resistant microorganisms: control of vancomycin-resistant enterococci. Pediatr Clin North Am.

[25] Smith PW, Bennett G, Bradley S, Drinka P, Lautenbach E, Marx J (2008). SHEA/APIC Guideline: Infection prevention and control in the long-term care facility. Am J Infect Control.

[26] Tresoldi AT, Cardoso LGO, Castilho GV, Dantas SRPE, von Nowakonski A, Pereira RM (2006). Low prevalence of vancomycin resistant enterococci colonization in intensive care patients in a Brazilian teaching hospital. Braz J Infect Dis.

[27] Maschieto A, Martinez R, Palazzo ICV, da Darini AL (2004). Antimicrobial resistance of *Enterococcus* sp. isolated from the intestinal tract of patients from a university hospital in Brazil. Mem Inst Oswaldo Cruz.

[28] Akturk H, Sutcu M, Somer A, Karaman S, Acar M, Unuvar A (2016). Results of four-year rectal vancomycin-resistant enterococci surveillance in a pediatric hematology-oncology ward: From colonization to infection. Tjh.

[29] Suzuki H, Hase R, Otsuka Y, Hosokawa N (2017). A 10-year profile of enterococcal bloodstream infections at a tertiary-care hospital in Japan. J Infect Chemother.

[30] Corso A, Faccone D, Gagetti P, Togneri A, Lopardo H, Melano R (2005). First report of VanA *Enterococcus*
*gallinarum* dissemination within an intensive care unit in Argentina. Int J Antimicrob Agents.

[31] Eshaghi A, Shahinas D, Li A, Kariyawasam R, Banh P, Desjardins M (2015). Characterization of an *Enterococcus*
*gallinarum* isolate carrying a dual vanA and vanB cassette. J Clin Microbiol.

[32] Pan SC, Wang JT, Chen YC, Chang YY, Chen ML, Chang SC (2012). Incidence of and risk factors for infection or colonization of vancomycin-resistant enterococci in patients in the intensive care unit. PLoS ONE.

[33] Worth LJ (2014). Vancomycin-resistant enterococci in patients with hematological malignancy: curbing an endemic pathogen. Leuk Lymphoma.

[34] Papadimitriou-Olivgeris M, Drougka E, Fligou F, Kolonitsiou F, Liakopoulos A, Dodou V (2014). Risk factors for enterococcal infection and colonization by vancomycin-resistant enterococci in critically ill patients. Infection.

